# Molecular Characterization and Landscape of Breast cancer Models from a multi-omics Perspective

**DOI:** 10.1007/s10911-023-09540-2

**Published:** 2023-06-03

**Authors:** Mylena M.O. Ortiz, Eran R. Andrechek

**Affiliations:** 1 Genetics and Genomics Science Program, Michigan State University, East Lansing, MI USA; 2 Department of Physiology, Michigan State University, 2194 BPS Building 567 Wilson Road, East Lansing, MI 48824 USA

**Keywords:** Mouse models, Modeling systems, Sequencing, Gene expression, Integrated analysis, Cancer subtypes

## Abstract

Breast cancer is well-known to be a highly heterogenous disease. This facet of cancer makes finding a research model that mirrors the disparate intrinsic features challenging. With advances in multi-omics technologies, establishing parallels between the various models and human tumors is increasingly intricate. Here we review the various model systems and their relation to primary breast tumors using available omics data platforms. Among the research models reviewed here, breast cancer cell lines have the least resemblance to human tumors since they have accumulated many mutations and copy number alterations during their long use. Moreover, individual proteomic and metabolomic profiles do not overlap with the molecular landscape of breast cancer. Interestingly, omics analysis revealed that the initial subtype classification of some breast cancer cell lines was inappropriate. In cell lines the major subtypes are all well represented and share some features with primary tumors. In contrast, patient-derived xenografts (PDX) and patient-derived organoids (PDO) are superior in mirroring human breast cancers at many levels, making them suitable models for drug screening and molecular analysis. While patient derived organoids are spread across luminal, basal- and normal-like subtypes, the PDX samples were initially largely basal but other subtypes have been increasingly described. Murine models offer heterogenous tumor landscapes, inter and intra-model heterogeneity, and give rise to tumors of different phenotypes and histology. Murine models have a reduced mutational burden compared to human breast cancer but share some transcriptomic resemblance, and representation of many breast cancer subtypes can be found among the variety subtypes. To date, while mammospheres and three- dimensional cultures lack comprehensive omics data, these are excellent models for the study of stem cells, cell fate decision and differentiation, and have also been used for drug screening. Therefore, this review explores the molecular landscapes and characterization of breast cancer research models by comparing recent published multi-omics data and analysis.

## Introduction

**Fig. 1:**
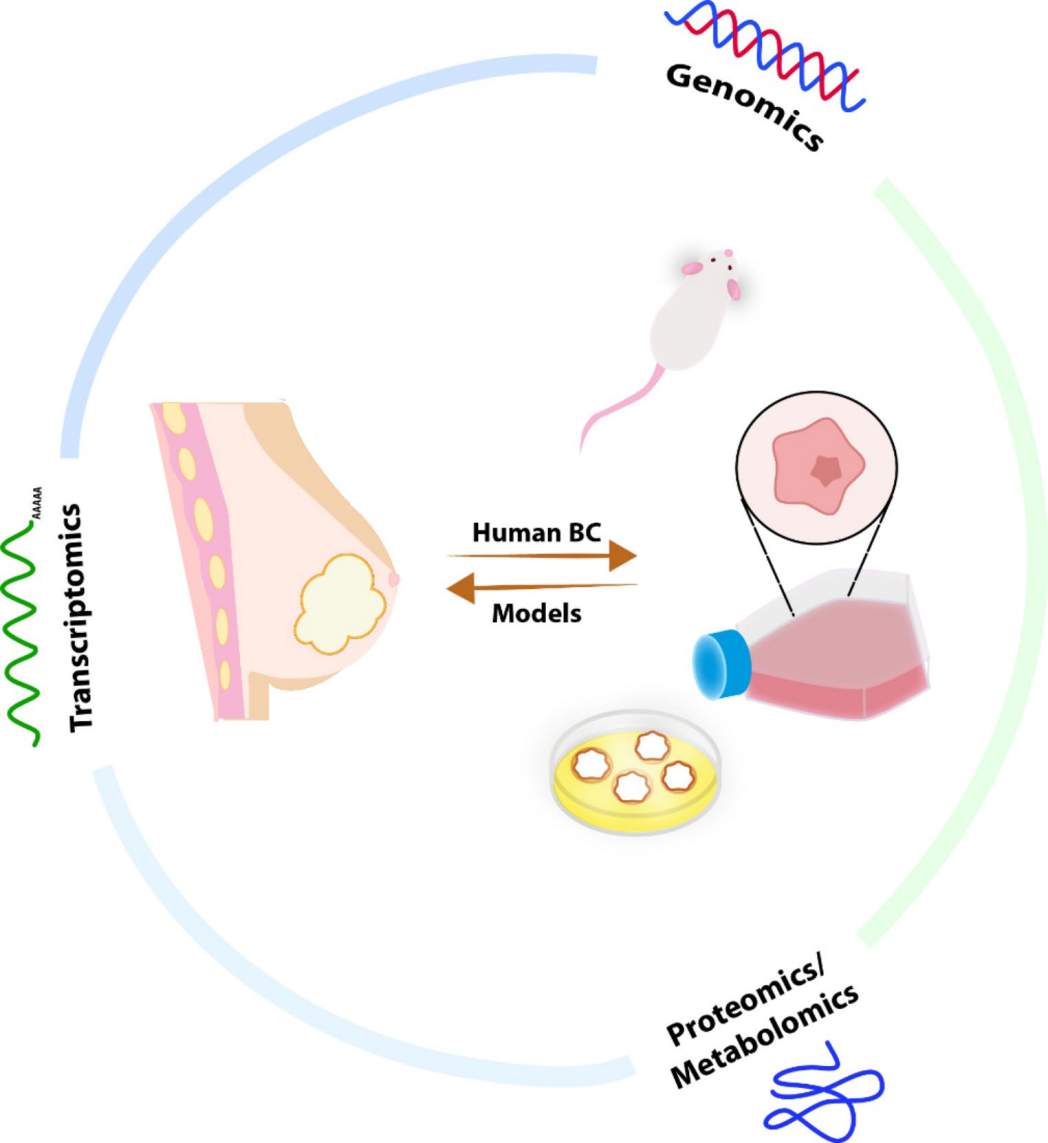
Multi-omics techniques have been uncovering the intrinsic features and parallels between human breast cancer and research models

Breast cancer currently represents 30% of new cancer cases in American women *(American Cancer Society, 2022)*. Therapy is dictated by stage and individual characteristics of each cancer with striking differences in the major subtypes. Given the differences between subtypes of breast cancer, choice of an accurate breast cancer model is essential. Due to the high genomic, transcriptomic, and proteomic landscape heterogeneity of human breast cancer, choosing the optimal model to address breast cancer research can be challenging [1]. Currently, a variety of in vivo and in vitro research models are available, ranging from cell lines, 3D cultures, murine models, mammospheres, patient derived xenografts (PDX) to patient derived organoids (PDO). With the rapid progress of omics technologies, researchers have been now gathering and cataloging information on the molecular mechanisms of both cancer and the research models used to study it [2]. Recently advances in machine learning have been making inroads and are poised to change precision medicine [3]. This review will examine the molecular characterization and landscape of breast cancer models from a multi-omic perspective (Fig. 1) with particular attention to how each system resembles human breast cancer subtypes and the advantages of each.

### Multi-omics Analysis of Human Breast Cancers

#### Transcriptomics in Human Breast cancer

Prior to the advent of microarrays, breast cancer classification was determined by the status of markers such as BRCA, estrogen receptor (ER), progesterone receptor (PR) and HER2. Diagnosis was refined by relying on the histological description and other tumor characteristics including metastasis and invasion of the lymph nodes. The initial description of a classification system using transcriptomics in a semi-supervised clustering analysis revolutionized the field and introduced the now familiar luminal A/luminal B / basal / HER2 / normal-like subtypes [4–6]. The initial set of classification genes was refined and the claudin low subtype was added [7, 8] and eventually was FDA approved to predict the subtype of breast cancer. Given the diversity of gene expression present within basal tumors, it was not surprising that subtypes were noted with differences in survival outcome [9]. Following this, the basal subtype was refined to contain 4 subtypes: two basal-like (BL1, BL2), a mesenchymal (M), and a luminal androgen receptor (LAR) subtype [10]. In 2012, a large comprehensive multi-omics study of 825 human breast cancer samples was published revealing novel molecular features of each subtype as part of The Cancer Genome Atlas (TCGA) project [11]. This revealed gene expression patterns that were characteristic of and augmented the intrinsic subtypes, from *ESR1, GATA3, FOXA1, XBP1 and cMYB*, in ER+/luminal-like subtypes to high expression of receptor tyrosine kinases like *FGFR4, HER1/EGFR* and loss of *PTEN* and *INPP4B* in the HER2 subtype.

The shift from microarrays to RNAseq has also allowed the development of RNA sequencing at the single-cell level. This has resulted in new dimensions of mammary gland development and breast cancer heterogeneity being uncovered. Lineage tracing of mammary epithelium in different development stages revealed cell populations and differentially expressed genes for each cell type according to the development phase, composing a new cluster of signature genes and shedding a light on cell fate decision [12, 13]. Although the specific timeline in which the mammary cell commits to a lineage (basal, luminal progenitor or mature luminal) remains an open area of investigation, it appears that embryonic cells are closely related to the basal population and commitment starts in early postnatal stages, finishing during puberty. Interestingly, intermediate populations arise, and population composition constantly changes during development.

In breast cancer, while bulk RNAseq was found to closely resemble single-cell profiling, particularly in the Luminal and HER2-enriched subtypes, the single-cell approach helped to define other characteristics important to the understanding of cancer biology [14]. Within tumors, mixed subtypes and cell composition of carcinoma and non-carcinoma cells, which includes mainly stromal (such as fibroblasts) and immune cells (i.e. tumor-associated macrophages and different phenotypes of T and B lymphocytes), are observed in different proportions. As an example, while Luminal subtype tumors are largely composed of carcinoma cells expressing high levels of ER and its canonical pathway genes, basal breast cancer is marked by immune infiltration, which likely contributes to the high heterogeneity of this subtype.

In addition to single-cell level work, spatial transcriptomics has allowed the complex interactions of human breast cancer, and the subsets of cells that make up a tumor to be examined. In a recent analysis, breast cancer was stratified into nine ecotypes (E1-E9) with the various cell populations defined by a single-cell derived subtype (SC) algorithm and then extended to bulk RNAseq data to partially associated it with the PAM50 intrinsic molecular subtypes [15]. Although a total of 9 major cell types have been identified, most ecotypes agglomerate cells from major cell lineages, i.e. epithelial, stromal, and immune cells, but in different cell states and with unique compositions of the last two. Importantly, the spatial organization of the cell states in discrete zones of the tumor suggests a role of the microenvironment in driving the zone phenotype, such as proliferative or mesenchymal-like. Different prognoses can also be correlated with the ecotype. For instance, E7 and E3, which are enriched in HER2_SC and HER2 tumors or basal_SC, cycling and luminal progenitor cells, respectively, have a worse 5-year survival. The complex interplay between cell types in this manuscript provides a unique glimpse of the complex interactions seen in cancer biology that were first seen through histology.

### Proteomics

It is well appreciated that the correlation between mRNA and protein abundance may vary greatly, emphasizing the importance of validating how well breast cancer mRNA subtyping is reflected in proteomics. Overall, unsupervised proteomics analysis of breast cancer tumors resembles the PAM50 subtypes for the basal-like, luminal A and normal-like, whereas Luminal B and HER2 + present mingled profiles, reflecting similarities between these two phenotypes [16]. Furthermore, highly proliferative subtypes (basal-like, Luminal B and HER2+) were found to have a greater correlation between transcriptome and proteome in comparison to the low-proliferative subtypes (normal-like and Luminal A). Proteomes from Luminal, HER2 + and basal-like contain an enrichment of E2F and MYC targets as well as G2M checkpoint proteins, however, basal-like tumors are distinguished by immune markers in special MHC class proteins. Increased proliferation and glycolysis, features of the Warburg effect, are notably observed in HER2 + and Luminal B subtypes. Just as was noted with gene expression for the basal subtype, proteomics and metabolomics can divide basal breast cancer into 3 subtypes: C1, enriched in sphingolipids and long-chain and unsaturated fatty acids, C2, presenting high metabolism of glutamate and carbohydrates and oxidation reaction, and C3, which metabolomics are more closely related to normal breast tissue [17]. The mRNA basal breast cancer subtype luminal androgen receptor (LAR) fits within the C1 subtype, while basal-like immune-suppressed (BLIS), immunomodulatory (IM) and mesenchymal-like (M) within the C2 and C3. In addition, LAR tumors are noted by the high activation of the ceramides pathway and levels of SP1, while BLIS tumors are abundant in NAAG, arising them as potential tumor-promoting metabolites.

### Genomic Alterations

Underlying the transcriptomic and proteomic characteristics are genomic alterations. An obvious example is the amplification of HER2 that results in overexpression and signaling. However, a more nuanced examination reveals that the amplification of HER2 includes other neighboring genes, with resulting gene expression alterations [18]. Since approximately 62% of gene amplifications result in elevated gene expression, this allows for copy number alterations to be predicted from gene expression data [19, 20]. Aside from predicting alterations, the TCGA project resulted in extensive sequencing data. As expected, a majority of tumors were noted to have p53 alterations with a substantial fraction also harboring PIK3CA mutations [11]. The TCGA data does permit a detailed view of events in specific subtypes. For example, the Luminal B subtype is overrepresented with ATM loss and Cyclin D1 and MDM2 amplification.

While the TCGA data and other large scale sequencing studies such as COSMIC [21] have revealed individual tumors with single nucleotide variations relative to the reference genome, many of them remain variants of unknown significance. Other mutants that result in frameshifts and missense mutations can have additional information about their potential importance gained from combining this mutation data with the cancer dependency atlas [22]. This integrative approach is essential in combining the multiple data streams with a combined analysis of transcriptomic and sequence data being a much more powerful analysis.

### Summary of the multi-omic Analysis of Human Breast cancer

Recent publications of multi-omic analysis highlight the utility of data intensive biology, but also illustrate the complexity of analysis. A recent manuscript detailing the workflow illustrates the effort required for a single patient with multiple biopsies, but also reveals how the multiple data streams can be successfully integrated [23]. Classification of breast cancer tumors according to the classic PAM50 mRNA subtypes reflects the transcriptomic landscape, but interrogating each of these subtypes from a multiomics perspective reveals novel molecular features, helping to understand their heterogeneity [11]. For instance, stratifying the basal-like subtype into new groups according to the different nuances of proteomics and transcriptomics landscapes emphasizes the complexity of these cancers [10, 17]. Alongside, unique genomic alterations have been found in normal-like, basal-like, luminal, HER2+, and claudin-low subtypes [11, 19–21]. These observations provide us with a comprehensive spectrum of breast tumors. Hence, acknowledging the intrinsic subgroups and underlying molecular features can enlighten how cancer behavior and response to therapy are dictated.

**Fig. 2:**
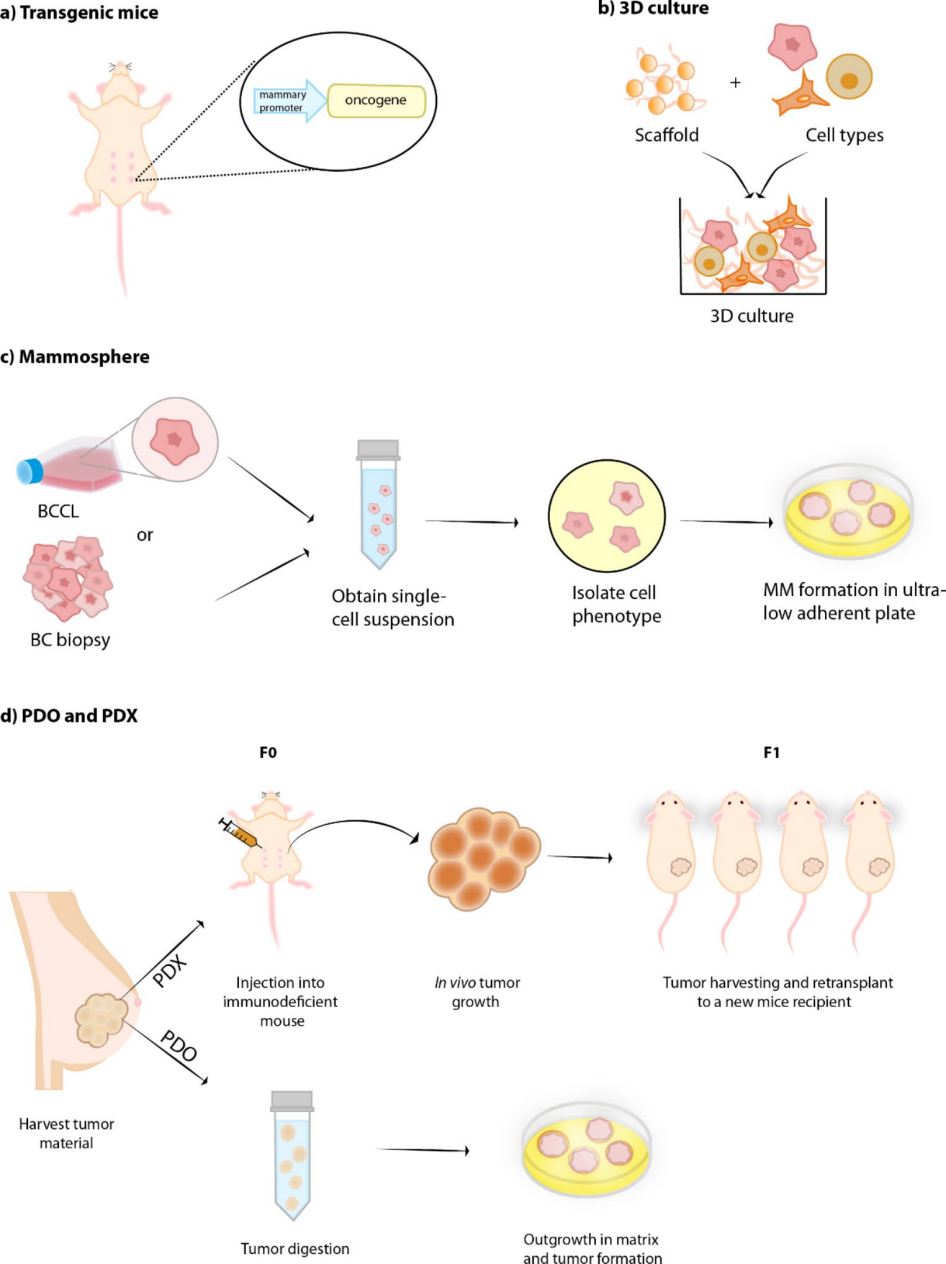
Simplified representation of breast cancer (BC) models generation. (**a**) Transgenic mice: one common approach for BC genetically engineered mice model generation is to overexpress an oncogene driven by a specific promoter targeting the mammary gland, such as MMTV. (**b**) 3D culture: the combination of a supporting scaffold (scaffold-dependent model), such as hydrogels and inert matrices, and different cell types allow cell growth and cell-extracellular matrix and cell-cell interactions. (**c**) Mammospheres (MM): these spheroids can be originated either from breast cancer cell lines (BCCL) or from BC biopsy. A single-cell suspension is obtained from the material, cell phenotypes are sorted for stem and progenitor cells, followed by culture in an ultra-low adherent surface for MM formation. (**d**) Patient-derived xenograft (PDX): tissue fragments from patient’s tumor are directly transplanted onto the immunodeficient mice heterotopically or orthotopically, with no need of an in vitro preparation step (F0). Once tumor reaches appropriate size, it can be dissected and expanded by reimplanting it onto another mice recipient (F1). The tumor expansion can go on for multiple generations (Fn). Patient-derived organoid (PDO): tissue fragments from patient’s tumor are digested and cultured in a 3D extracellular matrix hydrogel, giving rise to organoids

In order to study human breast cancer, there are a number of model systems. Each system (Fig. 2), has strengths and weaknesses and are usually suited for particular experiments. However, recent omic data has allowed a more detailed examination of the suitability of these model systems.

### Murine Models

#### Subtypes of Mouse Models

The description of inbred strains of mice that were susceptible to mammary tumors after fostering pups [24] led to the discovery of mouse mammary tumor virus (MMTV) through an interesting series of discoveries, from MMTV to the genes misregulated at the integration sites [25, 26]. Modeling cancer in mice was revolutionized with the description of some of the first transgenic mouse models of breast cancer expressing well known oncogenes like Myc [27], Ras [28] and Neu [29] under the control of the MMTV glucocorticoid regulated promoter enhancer [30]. In addition to overexpression models, there were also a series of knockouts used to study breast cancer with early knockouts often suffering from embryonic lethality [31–33] or in a surprising finding for BRCA1 heterozygous mice, lacking tumor formation [34, 35]. Development of independent MMTV-Cre transgenics in the Hennighausen [36] and Muller [37] labs allowed for tissue specific control of gene expression, including expression of the activated Neu allele under the control of the endogenous promoter [37]. Today, the design of mouse models of breast cancer has evolved to be induced / de-induced through tet-on systems [38], can be lineage traced [39, 40], can have multiple transgenes with an IRES system [41] and numerous other technical advances that allow for precise questions to be addressed. These advances allow for many aspects of tumor biology to be addressed, from metastasis in widely used models like MMTV-PyMT [42] to tumor heterogeneity [43, 44]. Indeed, the heterogeneity is present across many mouse models [45] and while this mimics human breast cancer, at the same time it confounds the analysis as the overexpression of an oncogene is too reductionist of an approach. Hence, understanding the molecular landscape of murine models and their variety of tumors becomes crucial.

### Histological Subtypes

Early in the analysis of mouse mammary tumor models it was noted that there were specific histological characteristics that were associated with expression of given oncogenes. This was encapsulated in a review of various initiating oncogenes and the physical characteristics of their tumors [46]. While the hypothesis that there are defining histological subtypes associated with specific oncogenes holds, an analysis of larger number of samples revealed the heterogeneity within individual models. Here a dominant histology was noted with other subtypes arising in a smaller population [43, 44]. More recently, it was noted that there were specific gene expression programs associated with the various subtypes that were predictive in nature [45]. While human breast cancer is not associated with the same subtypes noted in the mouse models, there are patterns that do hold, including an epithelial to mesenchymal transition (EMT). However, the EMT noted in primary tumors in mouse models is not associated with metastasis [44], a marked difference from the prevailing opinion in human breast cancer [47]. The examination of the histological subtype is a key component in the analysis of mouse mammary tumor models as they are reflected in the gene expression patterns that were noted to occur [44].

A key difference between mouse models and human breast cancer is noted at the immunohistochemical level. A substantial fraction of human breast cancers stain positively for the ER and the PR but there is a paucity of mouse models that are ER or PR positive. Stat1 deficient mice develop ER positive mammary tumors [48] and the MMTV-PyMT tumors [42] begin as ER positive but transition to ER negative during tumor progression [49], but other genetically engineered mice are lacking in this regard. This is poised to change with the development of lines such as the activated ESR1 mice [50] that can be interbred with other GEMs, which should allow for the development of ER positive mouse models of breast cancer.

### Transcriptomics in Mouse Mammary Tumors

Transcriptomics profiles of murine mammary tumor models have now been detailed by a number of groups. Aside from individual characterization of models, three studies have completed larger cohort analyses spanning many mouse models. This includes key comparisons of mouse models to human breast cancer [51–53] that revealed the extent of similarity of the models to the human disease. Removal of batching effects and interspecies differences allowed for the direct comparison of human breast tumors and murine tumors. This allowed the identification of common features across species and association with the human breast cancers subtypes (Table 1).

**Table 1:** Association of murine models with the human breast cancer samples. * Distinction between Luminal A and Luminal B not provided by authors. Note that not all murine models currently available are shown in this table. Only the most representative subtypes of each model published in the literature was assigned here. MMTV = Mouse mammary tumor virus promoter / enhancer, WAP = Whey acidic protein promoter

Murine Model	Human Breast Cancer Subtype					
	Basal like / TNBC	Luminal A	Luminal B	HER2 Enriched	Normal Like	Claudin Low
MMTV_PyMT				X		X
MMTV-Neu		X		X		
MMTV-Myc (EMT)	X					
BRCA1+/-; p53+/-; IR	X					
MMTV-Cre BRCA1^fl/fl^ p53+/-	X					X
MMTV-Wnt1	X				X	
Medroxyprogesterone DMBA induced	X					X
C3(1)-Tag	X					X
WAP-Myc		*	*	X		X
WAP-Int3		*	*			
PI3KCA-H1047R		*	*			
Stat1 -/-		*	*			
WAP-T121				X		X
MMTV-LPA					X	X
P53+/-; IR	X					X

There was high agreement amongst signature genes of the basal-like subtype, which include *Laminin gamma 2, Keratins 5, 6B, 13, 14, 15, TRIM29, c-KIT and CRYAB*, was found in the models harboring *BRCA, p53* and *Rb* deficiency (Brca1+/-, ;p53+/-;IR, MMTV-Cre;Brca1Co/Co; p53+/-, MMTV-Wnt1, and a few DMBA-induced) [52]. The MMTV-Wnt1 model can also overlap with normal breast-like, while the MMTV-Neu tumors unexpectedly associate with the Luminal A subtype instead of the HER2 subtype [53] While one analysis suggested that MMTV-PyMT and WAP-Myc models were similar to HER2+/ER- and/or luminal tumors [52] a more nuanced examination, including the histological subtypes, suggested that Myc and PyMT tumors with EMT histology resembled the gene expression markers of the claudin-low subtype [51], highlighting the heterogeneity of tumors that can be driven by these oncogenes.

The MMTV-Myc model can give rise to distinct tumor histological subtypes, each associated with a gene expression cluster [44]. Interrogation of the transcriptomic data with cell signaling signatures revealed histological subtypes that activate specific pathways. For example, papillary and microacinar tumors both activate E2F1 and Myc pathways, but the papillary has activation of Stat3 while microacinar has elevated B-cateninin signaling. EMT was noted for high expression of the Ras pathway, but low expression of Myc. Given the dominance of KRas over Myc [54] the induction of KRas mutations in the MMTV-Myc model and the subsequent development was not surprising. The mouse EMT subgroup appears to resemble human data more closely, and elevation of EMT signature is found in the triple negative subtype, which is also associated with greater metastasis potential.

The histological disconnect between ER positive human breast cancer and the mouse models that fail to develop ER positive tumors was again observed in the transcriptomic data [52, 53]. However, this analysis did identify an association of human ER positive luminal tumors with the WAP-Int3 (Notch4) [55], MMTV-Pik3ca-H1047R [56] and Stat1^−/−^ models.

### Whole Genome Sequencing of Mouse Mammary Tumors

Relative to the models analyzed through transcriptomics analysis, there is a dearth of whole genome sequencing data for mouse mammary tumor models. Indeed, a search of the literature reveals whole genome sequence data for only four models [18, 57, 58], including the NRL-PRL model which has elevated prolactin [57], p53 null and the MMTV-Neu [59] and MMTV-PyMT [42] models. The Prolactin model identified both mutations and copy number alterations in Kras and transcriptional analysis confirmed activation of the pathway [57]. Not surprisingly, the p53 null model resembled the basal breast cancer subtype and the WGS data was used in conjunction with transcriptomic data to identify new therapeutic approaches [58].

Compared to human tumors, both the MMTV-Neu and MMTV-PyMT murine models have a lesser mutation burden. Copy number alterations (CNA) are markedly predominant in MMTV-Neu and they are likely associated with increased activity of PI3K/AKT/MTOR signaling pathway. In the MMTV-Neu tumors, CNA in the 11D locus, a chromosomal region homologous to the human 17q21.33, leads to amplification of the genes Collagen type 1 alpha 1 *(COL1A1)* and Chondroadherin (*CHAD)*. This is a genomic event frequent in 8% of human breast cancers, of which 25% are HER2-enriched, and depletion of these two genes impacts migration and the ability to form tumors[18]. Interestingly, over 80% of MMTV-PyMT tumors have a V483M mutation in *Ptprh*, a phosphatase targeting EGFR and other kinases. Mutation of *Ptprh* results in phosphorylation of EGFR and upregulation of downstream pathways at a transcriptomic level [60].

Together each of these models illustrate the power of integrated sequence and transcriptomic analysis with each model system having accumulated genomic events that result in transcriptional changes. Other studies have employed exome sequencing to identify mutations in other model systems, including other backgrounds of MMTV-PyMT [61], Trp53 and BRCA2 deficient strains [62].

### Epigenomics of Mouse Mammary Tumors

Epigenomic regulation studies in murine models of breast cancer are currently lacking, hence a comparison with human breast cancers from this perspective is not accurate. Nonetheless, few conclusions can be drawn based on the epigenetic information available in the literature. In the *c-Neu (Erbb2/HER2)* cancer models driven by the MMTV promoter, overexpression of the oncogene and tumor development is owned to promoter demethylation, an event that happens in early stages of development [63]. Genome-wide chromosomal losses in these animals, such as loss of heterozygosity at chromosome 4 or 15, are also likely to be associated with a non-identified epigenetic mechanism [64]. Epigenetic reprogramming can also be influenced by diet: calory restriction was shown to preserve ER expression in MMTV-neu transgenic mice by differently methylating CpG islands within or in the flanking regions of the *ESR1/ESR2* genes [65]. In overweight and obese mice, expression of the methylation enzyme *DNMT1* is increased compared to lean/calory restricted mice, hence it is possible that other genes are being targeted. Besides epigenetic regulation at the gene level, energy balance is linked to histone modifications [66]. Activation of mTORC1, a regulator of cellular biogenesis which activity is coupled with mitochondrial energy generation, drives translation of Polycomb Repressive Complex 2 (PRC2) proteins, which in turn transcriptionally silence genes by methylation of H3K27 histones. Similarly, methylated loci enriched in PRC2 targets were identified in MMTV-PyMT mice [67]. Tumor progression towards a malignant phenotype shifts the global methylation degree towards a hypermethylated state. As a parallel with human breast cancers, different methylation states also accompany the human intrinsic subtypes, and the luminal B subtype is marked by enriched methylation among all [11, 68].

### Proteomic and Metabolomic Analysis of Mouse Mammary Tumors

The first broad survey of metabolomic heterogeneity in murine models (MMTV-PyMT, MMTV-PyMT-DB, MMTV-Wnt1, MMTV-Neu, and C3(1)-Tag) was demonstrated through principal component analysis (PCA) [69]. Murine tumors are overall enriched in glucose and amino acid metabolism, enhanced production of phospholipid precursors, TCA cycle intermediates, and cholesterol uptake compared to normal mammary tissue, which provides the energy and metabolic precursors necessary for tumor modulation and metabolic reprogramming. The Wnt1-induced tumors are the ones that harbor the most diverse and discrepant metabolomics compared to all other models, these tumors present active amino acid metabolism, but low levels of free and long-chain fatty acids, lysolipids and phospholipids, along with affected metabolic biosynthesis. Similarly, C3(1)-Tag tumors are generally low in fat metabolism and overall metabolites, but high in glycogen compounds. Gene expression regulation accounts for most of the metabolic changes observed in this model and genes related to increased proliferation, division, and nucleotide synthesis are upregulated. In comparison to MMTV-PyMT, MMTV-PyMT-DB, a mutated form of PyMT that prevents PI3K activation, differs by high levels of glutathione, glycogen storage, and long-chain fatty acids, and low levels of eicosanoids and glucose metabolism, suggesting that in PyMT active glycogen synthesis is taking place and in PyMT-DB there is reduced fat breakdown. MMTV-Neu tumors share some metabolic profile similarities with the PyMT-DB and are distinguished by lipid metabolism, with prominent activation of the inositol pathway, and amino acids metabolism.

### Breast cancer cell Lines

Breast cancer cell lines are widespread in cancer research and basic information including origin, hormone status and limited clinical parameters are often provided for each line. MCF7, MDA-MB-231 and T47D are the most used breast cancer lines and with the advance in technology and publicly available omics datasets, such as the Cancer Cell Line Encyclopedia (CCLE) which provides a compilation of gene expression, CNV`s and parallel sequencing of over 1000 cell lines, it is now possible to globally characterize these models and gain insight into drug response, pharmacogenomics and how accurate they mirror human breast cancers [70, 71]. There are clear similarities between the cancer cell lines and breast cancer, with shared mutations in key genes such as p53, RB and PI3K [72] and transcriptional investigations that have revealed that the cell lines largely cluster into the basal and luminal groups [73]. It is critical to note that breast cancer cell lines are heterogeneous as seen in barcoding [74] and single cell analysis [75], with functional differences arising in the clones, especially with regard to metastatic potential [74]. This heterogeneity is reflected in signature breast cancer receptors and biomarkers expression: although some of the prominent biomarkers from each of the PAM-50 subtypes are present across the 32 breast cancer lineages analyzed by single-cell sequencing, expression of clinically relevant genes, such as HER2, can vary greatly within a cell population [75]. As additional evidence, different cell lines of origin were found to be present within the same cluster, as observed for the luminal-like cell clusters. Curiously, TNbreast cancer clusters were found to group cell lines from the same line of origin. It cannot be understated that clonal heterogeneity within individual lines is a key consideration when designing CRISPR knockout studies and choosing to take a clonal or population-based approach.

However, while breast cancer cell lines are readily used and easily adapted to studies in the laboratory, there are significant concerns about their utility as a breast cancer model system. Increasingly studies have demonstrated poor similarity between breast cancer cell lines and human tumors [76–78]. Each cell line has accumulated specific genomic alterations of its own along with some shared features with primary breast tumors. For instance, cell lines have more mutation burden and copy number alterations, which are present across all chromosomes, compared to tumor specimens [73, 79]. Mutation burden and the genes in which these somatic mutations occur can also be significantly different, the majority of genetic alterations present in different lineages of breast cancer cells either do not overlap with human metastatic specimens or mutation frequency is significantly lower in one compared to the other, meaning that only a few cell lines can recapitulate important genes involved in breast cancer progression and metastasis. Among 75 unique mutated genes identified in metastatic tumors from the MET500 dataset, 9 of them are not present in any of the over 1000 cell lines from the Cancer Cell Line Encyclopedia, including *ESR1*, which is a metastasis driver and the 5th most altered gene preceded by *TP53, PIK3CA, TTN* and *OBSCN*. From the transcriptomic side, CCLE data reveals that MDA-MB-415 cells have the highest correlation with its tissue of origin and metastatic sites (liver and lymph node), and it is curiously the one that resembles Luminal A subtype the most [79].

Although RNA profile clustering from some cell lines with human breast cancer subtypes shows some conserved features, proteomic clustering does not seem to follow the same pattern and reveals an even weaker overlap with tumor samples [78]. For instance, BT483 and T47D harbor similar protein levels in human breast cancers, whereas MCF7 presents a moderate correlation [79]. The proteomic differences could be explained by interference of other cell populations residing within and in the tumor surroundings, conditioned in vitro growth, as well as different biological processes prioritized in vivo and *in vitro.* Furthermore, each of the cell lines analyzed (MDA-MB-231, MCF-7, SK-BR-3, JIMT-1, MCF-10 A) expressed similar protein profiles, suggesting the existence of a “core proteome” in tissue culture, but display a poor correlation between RNA and protein levels, highlighting that transcriptomics is not always translated into suitable protein biomarkers. This variation had been previously reported for strong to low or no concordance between breast cancer cell line microarray data and western blot analysis [73]. Focusing on the metabolomic portion of the proteomic profile reveals that it is unique to each cell lineage, and it is seemingly not associated with genomics or gene expression alteration, nor the breast cancer subtype [80, 81]. Instead, it can be affected by the cell phenotype, time parameter, and oxygen supply, with hypoxia accounting for greater variability compared to normoxic conditions [81].

Murine breast cancer cell lines and their association with human breast cancers and the PAM50 subtypes have also been explored [82]. From a panel of 12 metastatic murine mammary tumor cell lines (4T1, 6DT1, D2A1, E0771, EMT6, F311, HRM-1, M6, Met-1, MVT1, r3T, and TS/A-E1), commonly mutated genes in human breast cancers were found to be present, including *PIK3CA* and *TP53.* In terms of CNV, only minor overlaps with the CNVs from human breast cancers were noted and were complemented with transcriptomic clusters. In addition, when modeling the human breast cancer subtypes, the majority of lines were predicted to resemble the Luminal A subtype, whereas E0771 and D2A1 resemble Luminal B.

From the epigenomics perspective, breast cancer cell lines from the same intrinsic molecular subtype share a chromatin pattern that is distinct from the others, as evidenced by analysis of the epigenetic markers H3K36me3 (activation) and H3K27me3 (repression) [83, 84]. Active transcription-associated states and enhancer states compose the majority of this commonality, whereas repressive states are more enriched in non-malignant cells [84]. In basal-like cell lines, hypomethylation of CpG sites is more expressive and it is associated with gene regulation of this aggressive phenotype [83]. Furthermore, a total of 58 genes were identified to be epigenetically regulated across a panel of 45 cell lines [85]. Some of them, such as COL1A2, TOP2A, VAV3, CDKN2A, and TFF1, have validated roles in tumor development.

When it comes to overlapping features with human breast cancers, the genome-wide DNA methylation pattern of cell lines and primary tumors resemble one another and highlight an evolutionary ancestry [86, 87]. However, crucial differences are also present. A striking example is the B-CIMP-associated loci, in which methylation is close to absent in ER- tumors, but it is found moderately to highly methylated in ER- cell lines [86]. In contrast, luminal-like triple-negative cell lines share the least number of features with their respective primary tumor on both methylation pattern and gene expression profile.

Although breast cancer cell lines and human breast cancers share some genomic and transcriptomic landscape, they fail to capture many molecular features of human breast cancer phenotypes or from their progenitor cells after several passages. Moreover, the tumor microenvironment cannot be reproduced in two-dimensional cell culture, individualized therapy screening is labor-intensive and representation of certain breast cancer subtypes, such as HER2-enriched, claudin-low, and normal-like, is restrained by the lack of an accurate breast cancer cell line options, increasing the challenge to address characterization and therapeutic strategies in vitro and call upon alternative models for such purposes [88, 89].

### Patient-derived Organoids and Xenografts

Organoids and patient-derived xenografts (PDX) are two promising alternatives to two-dimensional cell culture and animal studies that preserve the omics, pathophysiology, and intrinsic features of human breast cancers at a higher degree. Indeed, the initial characterization revealed both histological and gene expression similarities between PDX and the tumors they originated from [90]. While PDXs are human tumor samples grafted into immunodeficient mice, organoids are a 3D result of long-term culture of human cancer tissue resection, and both models have been often used to improve drug screening and therapeutics [91, 92]. Both models mirror the inter and intra-heterogeneity of human breast cancers, however, only PDX can simulate the tumor-stroma interactions. Organoids, on the other hand, have a faster expansion, allow high-throughput drug testing and genetic manipulation but are only viable for cells of epithelial origin when using adult stem cells, a limitation that can be circumvented by employing pluripotent stem cell-derived organoids [93, 94]. Broad characterization and annotation of normal mammary epithelium, cancer-prone and breast cancer patient-derived organoids (PDO) have been completed using either histology, gene expression profile, genome sequencing, and drug testing, revealing their ability to preserve heterogeneity and consistent features with their progenitors, such as molecular landscape, histological subtype and hormone receptor biomarkers status, even after continued passage number [95–97].

Although PDX are more laborious to establish and analyze by bioinformatics due to mouse genome interference and often lack matching patient germline and tumor omics information, good progress has been made to catalog and characterize these models for various cancer types [98]. Like PDO, PDX maintain tumor architecture [90] and inter and intra-tumor heterogeneity even after passages [99], have elevated genomic and transcriptomic similarity with their matching tumor tissue and are a cleaner representation of the tumor fraction, which can contribute to higher variant allele fractions compared to the sample they are derived from [98]. The majority of PDX samples were initially of the basal subtype, but significant strides have been made in obtaining tumors from other PAM50 subtypes.

Models to examine metastasis have been limited due to the technical challenges associated with detection of single cells in secondary sites [100]. PDX models coupled with single cell RNAseq circumvented these limitations to reveal that metastatic cells had different enriched pathways, metabolomics and transcriptomics profiles (303 different expressed genes) in cells from primary tumors and metastasis, and 116 upregulated genes in the latter, including heat shock proteins and cytokeratin genes, *ACTG2*, a gene involved in cell motility, and other genes which roles are lesser explored in metastasis progression, including *CKB, NME1, ASHA1, NOP16*, *S100A16* and *PHLDA2*, a gene associated with increased relapse in basal-like cancers. Metastatic cells are bioenergetically shifted from glycolysis (observed in primary tumors) to mitochondrial metabolism/oxidative phosphorylation (OXPHOS), and the metabolites that fuel or are intermediates of OXPHOS and the citric acid cycle (fumarate, malate, succinyl carnitine, fatty-acid metabolism and amino acids metabolism intermediates) are increased in those.

Despite the advantages to PDX and PDO model systems, there are still caveats. One of the primary concerns remains that the immune system is critical in tumor development and metastatic progression and the PDX models are generated in an immune deficient recipient mouse. Thus, care must be taken in experimental design when choosing a PDX or PDO model.

### Mammospheres and 3D Cultures

Tumor heterogeneity is not only a result of genetically diverse cell clones and clonal evolution but also from epigenetic modulators that allow transition, sometimes reversible, between stem, progenitors and differentiated cell phenotypes [101, 102]. A small population of stem-like cells with self-renewal potential is present in cancers, and they can give rise to heterogeneous tumors, promote invasion and metastasis [103]. Therefore, it is crucial to recapitulate cancer stem cells (CSC) in a research model and mammospheres accomplish this task by propagating CSC *ex-situ* mimicking the tumor micro niche. Mammospheres can be generated from tissue samples, metastases, or cell lines with cancer stem cell properties by maintaining and enriching these populations of cells [104, 105]. The generation of a 3D culture system is achieved spontaneously by culturing single cells in a non-adherent, serum-free environment, and the continued passaging of established MM can contribute to increasing the number of CSCs. Limiting dilution transplants into the mammary fat pad are used to assess CSC self-renewal, however conclusions drawn from these models must be taken with caution due to varied cell compositions that can arise after mammosphere establishment with potential concerns about clonal origin [106].

Different from 2D or ex vivo cultures, culturing cells in a 3D assay simulates the microenvironment by providing a rich extracellular matrix. Due to the importance of these models, 3D cultures of breast cancer cell lines grown in a Matrigel/hydrogel-collagen have been characterized structurally and biologically and assessed for chromatin interactions and differential reprogramming [107, 108]. Gene expression profiling of 14 breast cancer cell lines covering different breast cancer subtypes helped to identify a set of genes differentially expressed in these cells when cultured in 3D and 2D. This demonstrated that the number of differentially expressed genes was more expansive when comparing the breast cancer subtypes than the culture conditions.

Together, these findings give insights into the range of morphological and biological modifications that the cells undergo during adaptation from monolayers to three dimensional assays. In addition, these models open alternative doors for drug screening, characterization and role of stem/progenitor cells in breast cancer initiation and progression, understanding of cell-lineage differentiation, cell signaling and metastasis, and modulation by the microenvironment.

### Machine Learning Models

Although a focused examination of machine learning is beyond the scope of this review, it is worth noting the impact that computational models are adding to the existing omics data. Machine learning is as a combined technique sustained on mathematical algorithms to train computers to perform a complex task, such as accurate predictions [109]. Multiple algorithms with reasonably successful rate have been recently developed to predict breast cancer subtype classification based on the exome, RNA-seq and probe selection [110–114]. Further, breast cancer risk and survival can be refined by combining genetic variants, demography and known risk factors in a machine learning approach [115–117]. This can then be extended to personalized drug response predictions by taking into consideration the multi-omics landscape [118]. Taken together, the combination of multi-omic data with machine learning has the potential to revolutionize precision medicine by accelerating clinical handling and innovative applications. However, this must be tempered by the inclusion of wet-lab biologists into the decision tree as too often pure machine learning approaches can identify non-biological parameters.

## Conclusion

Here we focused on exploring the molecular landscapes of commonly employed breast cancer models and their association with the PAM50 human breast cancer subtypes. Considering that breast cancer is a highly heterogeneous disease from many perspectives, genetically, transcriptionally, histologically, and in proteomics, and different drug responses are observed for each patient, it is imperative to choose a research model with caution. As general guidelines, model selection must consider the hypothesis to be addressed and how the intrinsic features of each model will affect the ability to test the hypothesis. Of note, some models present conflicting data and categorization or can fall into more than one subtype group due to intra-model heterogeneity. Hence, careful analysis of the model using publicly available omics and histology data can guide the choice of an appropriate model. Besides research papers on model characterization, databases such as COSMIC, cBio Portal, and Depmap Portal from CCLE provide an easy-to-use and interactive interface for non-computational biologists interested in visualizing omics information. With this in mind, in this review we attempted to shed light on the omic implications of each model to clarify and guide breast cancer research.
